# Value-adaptive clinical trial designs for efficient delivery of publicly funded trials - a discussion of methods, case studies, opportunities and challenges

**DOI:** 10.1186/s12874-025-02566-6

**Published:** 2025-06-05

**Authors:** Laura Flight, Alan Brennan, Stephen E. Chick, Martin Forster, Steven Julious, Puvan Tharmanathan

**Affiliations:** 1 Sheffield Centre for Health and Related Research (SCHARR), School of Medicine and Population Health, University of Sheffield, Regent Court, 30 Regent Street, Sheffield, S1 4DA UK; 2 INSEAD, Technology and Operations Management Area, Fontainebleau, France; 3 Department of Statistical Sciences ‘Paolo Fortunati’, University of Bologna, Bologna, Italy; 4 York Trials Unit, Department of Health Sciences, University of York, York, UK

**Keywords:** Value-adaptive clinical trial design, Cost-effective research, Health economics, Adaptive designs

## Abstract

**Background:**

Value-adaptive designs for clinical trials are a novel set of emerging methods for delivering greater value for clinical research. There is increasing interest in using them within publicly funded health systems. A value-adaptive design permits ‘in progress’ changes to be made to the trial according to criteria which reflect its overall value to the healthcare system, including the cost-effectiveness of the technologies under investigation, the cost of running the trial and the total health benefit delivered to patients. These trial designs offer the potential to explicitly balance the costs and benefits of adaptive clinical trials with the health economic benefits expected for populations that are affected by any subsequent health technology adoption decisions. They may also improve the expected value of learning from the budget that is spent within a trial.

**Main body:**

This paper introduces value-adaptive designs for publicly funded clinical trials. It discusses the idea of delivering ‘value for money’ in health technology assessment, what is meant by being ‘value-adaptive’ and the key features that characterise these designs. The methodology behind one kind of value-adaptive design – the value-based sequential model of a two-armed clinical trial proposed by Chick et al. (2017) – is described and illustrated using three retrospective case studies from the United Kingdom. The paper concludes by reviewing a range of perspectives provided by stakeholders, together with our own thoughts, on the practical opportunities and changes required for implementing a value-adaptive approach.

**Conclusions:**

Value-adaptive clinical trial designs offer the potential to align health research funding allocations with population health economic goals. Many of the systems required to deploy value-adaptive designs within a publicly funded health system already exist and, with increased application, experience, and refinement they have the potential to deliver improved value for money.

## Introduction

Healthcare systems are experiencing rapid technological change and their ability to evaluate the potential offered by new treatments is under increasing scrutiny. Clinical trials have traditionally focused on assessing patient-level clinical effectiveness. However, in recent years, publicly funded healthcare systems have become increasingly focused on estimating the value for money offered by new health technologies [1, 2]. The observation that clinical trials and health technology adoption decisions are typically driven by different metrics – clinical effectiveness on the one hand and cost-effectiveness on the other – suggests an opportunity for designing clinical trials in a way that incorporates both health-related and cost-based criteria. This is the crux of taking a *value-based* approach to designing a clinical trial. *Value-adaptive* designs aim to make the value-based approach to assessing health technologies ‘adaptive’, that is, to exploit the flexibility offered by adaptive trials [3] in a cost-effective manner, to align the current value-for-money trend in healthcare delivery with that of trial design.

This paper explores value-adaptive designs and summarises the results of recent research which applies a specific kind of value-adaptive design – a sequential clinical trial with two arms whose stopping rule is determined by value-adaptive criteria – within the context of publicly funded trials in a single-payer system. The value-adaptive approach places additional demands on a clinical trial’s data collection processes, because the costs of both the health technologies and the research process must be estimated, together with the size of the population of patients to benefit from the technology adoption decision [4–8]. As it is becoming increasingly common to measure the costs of treatments as part of health technology assessments, it is natural to ask whether taking a value-adaptive approach can improve the value for money of publicly funded clinical research.

We consider value-adaptive designs from the perspective of a large public funder such as the UK’s National Institute of Health and Care Research (NIHR). Alongside fulfilling their clinical responsibilities, the NIHR is directly involved in the design and running of health technology assessments (HTAs). The NIHR supports the delivery of novel, complex and innovative clinical trials, including adaptive trials (e.g., STAMPEDE [9], Randomised Evaluation of COVID- 19 Therapy RECOVERY [10]). Additionally, evidence from NIHR-funded studies is used to inform national clinical guidelines and HTA decisions for new and existing health technologies.^1^ The NIHR has prioritised improving the efficiency of clinical trials [11], stating that it is “keen to see the design, development and delivery of more efficient, faster, innovative studies to provide robust evidence to inform clinical practice and policy” [12].

Sect."Background"of this paper introduces the background to value-adaptive trials. Sect."Value-adaptive clinical trials"discusses a range of aspects of clinical trial design which could benefit from the value-adaptive approach, describes the methodology behind the value-based sequential design that is the focus of this paper and applies it to three retrospective case studies using data from UK clinical trials. Sect."Implications of taking a value-adaptive approach in publicly funded research"summarises the views of stakeholders including funders, clinicians, trial teams, the public and healthcare decision makers, as well as our own thoughts, on opportunities and changes required to adopt a value-adaptive approach in a publicly funded healthcare system such as the NHS.

This paper presents results from the EcoNomics of Adaptive Clinical Trials (ENACT project). ENACT was part of the NIHR’s Efficient Studies funding call (2019) for Clinical Trials Units (CTUs). The ENACT project team undertook a series of workshops with key stakeholders from across the NIHR on the potential use and implementation of value-adaptive methods in NIHR research. It also funded two of the retrospective case studies whose results are reported in this paper.

## Background

Taking a ‘value-adaptive’ perspective to designing a clinical trial means being both ‘adaptive’ and ‘value-based’. An-adaptive clinical trial analyses data as it accumulates over the course of the trial to inform changes which meet pre-determined objectives for the healthcare system and/or its funder. An adaptive design might offer the option to stop the trial earlier or run the trial longer than planned, or to maintain or change the ratio of patients allocated to its arms, according to how persuasive the accumulated evidence is at one of the trial’s ‘interim analyses’. An adaptive trial differs from what we refer to in this paper as a ‘fixed sample size’ trial, which recruits to a predetermined sample size, using fixed allocation ratios to the trial’s arms, and for which no changes are permitted in response to accumulating evidence. Adaptive designs have the potential to prevent patients from being needlessly allocated to unpromising treatment arms and to deliver the better treatment to patients sooner. They are becoming more common [3, 13, 14] and there now exists guidance on their operation, as well as discussion of the methodological challenges that they pose [15, 16].

Adaptive trials can be designed according to frequentist or Bayesian principles. The frequentist approach assesses accumulating evidence using hypothesis tests which meet predefined criteria for statistical significance and power. The Bayesian approach uses evidence from the trial to update a so-called ‘prior probability distribution’ for an unknown value of interest—such as the difference between the average efficacy of two or more health technologies—in the population of patients which meet the trial’s inclusion criteria.

In a ‘value-based’ adaptive clinical trial – which we refer to hereafter as a ‘value-adaptive’ clinical trial – changes to the way the trial operates are informed by estimates of the costs and benefits of the health technologies and potentially the costs and benefits of the trial itself. In the approach that we take in this paper, value-adaptive designs are Bayesian designs. We discuss this approach in Sect."Value-adaptive clinical trials".

One of the key requirements of taking a value-based approach is that patients’ health outcomes are valued in monetary terms. This permits the costs and benefits of the health technologies, as well as the costs of carrying out the trial, to be valued in a common metric. Valuing health outcomes in monetary terms is becoming increasingly common in the HTA literature [17]. For example, when a body such as the UK’s National Institute for Health and Care Excellence (NICE) assesses new health technologies, it typically estimates the additional Quality Adjusted Life Years (QALYs) gained for a patient receiving the new technology, compared to existing care, as well as the additional cost that is likely to be incurred by the NHS and social services in providing that technology [18]. NICE uses this information to inform a decision about whether the new technology is cost-effective and whether it should be approved for use in the NHS. Typically, a new treatment is considered cost-effective for the NHS if it is expected to deliver one additional QALY at a cost that is less than between £20,000 and £30,000 [18].^2^

Valuing health outcomes in monetary terms and comparing an accumulating estimate of cost-effectiveness with the cost of continuing the trial in its current form, versus changing it, permits a value-adaptive design to be informed by ‘value of information’ (VoI) methods. These methods have seen increasing use in UK HTAs in recent years [19], with published guidance on their use in non-adaptive settings [20, 21] and pre-trial cost-effectiveness modelling [22, 23]. The basic idea behind a VoI approach is that, as more patients are recruited into a trial, the estimate of cost-effectiveness becomes more precise, which reduces the risk of making an incorrect decision about which health technology is superior. However, the ‘value-added’ of information resulting from recruiting an additional patient declines as the trial’s sample size increases. As a result, the trial’s so-called ‘optimal’ sample size is determined when the expected benefit of recruiting another patient is equal to the expected cost of doing so.

Despite the interest in using VoI methods to design value-based fixed sample size trials [4, 19–21, 24], little work has considered extending the ideas to adaptive clinical trials. Flight et al. [24] found that cost-effectiveness criteria are not routinely incorporated into the design of adaptive trials, that adaptive trials rarely account for the costs of the research process and that, among those interviewed in a qualitative study, there was a perceived potential benefit to incorporate such issues into the design of future trials [25].

## Value-adaptive clinical trials

A value-adaptive approach can be applied to a range of features of clinical trials, some of which are summarised in Table 1. These include stopping a two-armed sequential trial using value-based criteria (discussed in Sect."The Bayesian value-based approach to designing an adaptive clinical trial").

**Table 1:** Features of clinical trials which can be made value-adaptive

Feature	Value-adaptive
Stopping a sequential clinical trial earlier than planned/running it later than planned	A value-based approach can be taken to stopping a clinical trial early or running it late according to value-based criteria. This problem was addressed by Chick et al. (2017) [7] and is the focus of Sect."The Bayesian value-based approach to designing an adaptive clinical trial"of this paper
Convert a fully sequential clinical trial into a group sequential design	Group sequential designs trials allow for multiple patients to be assigned to multiple treatments in one batch. Several methods exist to allocate multiple samples to a finite number of arms using Bayesian expected value of sample information [26–29]
Altering the fraction of patients that are allocated to the arms of the trial	Ahuja and Birge [30] use dynamic programming in a group sequential trial for Bernoulli (0–1) outcomes to adaptively vary the fraction assigned to each arm during each stage based on accumulating data. This aims to improve outcomes for patients in the trial as well as the probability of correctly selecting the best treatment and can be adapted to the valued-based setting by weighting outcomes by estimates of net monetary benefit
Changing the rate of patient recruitment to the trial	A value-based trial optimises the expected population INMB minus expected trial costs. If the recruitment rate is nonlinear (e.g., a trial manager may prioritise sites for opening because they are more likely to have a higher rate of successfully enrolled and retained patients), this information can be used to optimise the recruitment rate. Alban et al. [5] discuss this based on data from the ProFHER trial
Multi-arm and Phase II/III dose finding trials	Multi-arm trials may have correlated mean INMB for different arms. For example, in a dose-finding trial, similar dose levels may have a more similar mean INMB than very different dose levels. Adaptive allocation policies for highly sequential value-based multi-arm trials have been proposed and show promise in identifying optimal doses (as compared to precisely estimating the entire dose–response curve, even less-effective doses) [6, 8]
Incorporating the value accruing to patients who are part of the trial and those who are not	Chick et al. [7], Alban et al. [5] and Ryzhov et al. [31] discuss how the benefits of such patients can be incorporated into the value-based approach using so-called online learning techniques
Allow for a commercial firm and a public payer to collaboratively negotiate a price based on the estimated health value	Yapar et al. [32] study conditional approval schemes from a value-based trial framework and show how to adapt fixed-length value-based trials and to allow collaborative bargaining for price as a function of health value created
Accounting for precision medicine with predictive covariates as well as prognostic covariates	Alban et al. [33] illustrate how covariates can be accounted for to accelerate learning for precision medicine (with predictive covariates) or to reduce the variance of estimated performance (with prognostic covariates). This can nudge arm randomization decisions so that patients are more likely to receive treatments as a function of their covariates that likely result in a better outcome, a form of adaptive enrichment

Regardless of the precise feature being addressed, the value-adaptive design’s focus on estimating the cost-effectiveness of the health technologies under investigation—measured using the incremental net monetary benefit (INMB). This means that the patient-level costs of the technologies must be measured or estimated, in addition to the health outcomes. A HTA agency may also place a monetary valuation on a measured health outcome, using a societal maximum ‘willingness to pay’ for one unit of the health outcome, such as a maximum willingness to pay for one QALY. Furthermore, the value-adaptive design’s focus on the cost-effectiveness of the research process means that the fixed and variable costs of carrying out the clinical trial should be estimated, because they inform the decision about whether to adapt the trial as it progresses. Finally, the focus on the overall benefit of the trial to the healthcare system requires an estimate of the size of the population that is expected to benefit from the technology adoption decision that the trial informs. The next two sections examine these ideas in more detail.

### Estimating the cost-effectiveness of a health technology

We can measure the value that a health technology is expected to generate for a patient by converting its estimated health benefit – in the UK NICE the standard approach is to use the Quality Adjusted Life Year (QALY) – into a monetary measure [30]. In a clinical trial, this is achieved by calculating the average number of QALYs for patients who are treated with one of the health technologies of interest and multiplying the result by the maximum amount a decision maker, such as NICE, is willing to pay for one additional QALY (the so-called ‘willingness to pay’ (WTP) threshold). The estimated cost of providing the technology to a patient is obtained by calculating the average cost of treating the patients who have received that technology. These costs include the cost of the technology itself, the costs of administration, staff time and other resources.

These costs and benefits can be used to estimate the expected net monetary benefit (NMB) of the health technology for one patient. This is equal to the expected monetary benefit of the technology minus the expected cost:

$$ENMB=WTP\times EQALY-ECost.$$

One of the simplest trial designs compares two health technologies, such as a new health technology ‘A’ and one that is already in use (‘B’). We can estimate the expected incremental net monetary benefit (EINMB) from treating a patient with technology A instead of technology B (${EINMB}_{per\;patient}$), by subtracting the estimate of the *ENMB* for B from that of A:

1 $${EINMB}_{per\;patient}={ENMB}_{A}-{ENMB}_{B}$$

If there are no costs to the health service of switching from B to A, the new health technology A is adopted in preference to B if ${EINMB}_{per\;patient}$ in Eq. (1) exceeds zero. We can say that technology A is ‘cost-effective’, that is, it is expected to deliver a higher NMB than technology B. If $\star{{EINMB}_{per\;patient}}$ = 0, the technologies are expected to perform equally well and B is said to be cost-effective if ${EINMB}_{per\;patient}$ < 0. The value-based sequential design that we consider in Sect."The Bayesian value-based approach to designing an adaptive clinical trial"uses the cost and benefit data collected during the trial to estimate $\star{{EINMB}_{per\;patient}}$. A description of estimating the EINMB using data from a two-arm clinical trial is provided in Table 2.

**Table 2:** Estimating the expected incremental net monetary benefit using data from a two-arm clinical trial comparing technologies A and B

An illustration of an estimator $\left({\widehat{EINMB}}_{per\;patient}\right)$of the expected incremental net monetary benefit per patient $\left({\widehat{EINMB}}_{per\;patient}\right)$for the population of patients who meet the trial’s inclusion criteria is shown in Eq. (2). We assume we have a trial dataset with trial participants who received the new technology A and participants who received technology B. An estimate of the QALYs and costs for each treatment comes from the trial data. We use these to estimate the QALYs and the costs for patients expected to benefit from the technology once a technology adoption decision has been made. As the sample size increases, we become more certain of the expected net monetary benefit of each treatment and more certain as to which of the two treatments would be considered cost-effective by the public funder.

where $qalyA_i$ is the QALYs for participant $i$ receiving technology A in that arm of the trial and $\cos tA_i$ is that same participant’s NHS and social care costs. For a population of P patients who will be impacted by the technology adoption decision, the total, or population, expected incremental net monetary benefit is ${\widehat{EINMB}}_{pop}\;=\;P\;\times\;{\widehat{EINMB}}_{per\;patient}$ minus the cost of switching from the incumbent technology, B, to the new technology, A.

These methods measure cost-effectiveness at the level of the individual patient. A range of approaches can be used to calculate the total, or population, expected incremental value of the technology to the healthcare system. Under the assumption that the number of patients who will benefit from the technology adoption decision, *P*, is not related to the estimate of EINMB that results from the trial, population EINMB can be calculated by multiplying the per patient expected incremental net monetary benefit in Eq. (1) by *P*. One way to estimate *P* is to multiply the annual incidence of the condition that is being studied by the number of years over which the adoption decision is expected to apply. If P and EINMB are related – for example, if a higher EINMB is believed to lead to a larger size of the population to benefit, it is straightforward to model INMB as a function of P and then calculate the population benefit as E[P x EINMB(P)].

Finally, if the cost of switching from technology B to technology A is greater than zero, the population incremental benefit becomes E[P x EINMB(P)] – C, where C is the switching cost. Accounting for the total benefit provided by the health technology, and subtracting the cost of switching, therefore permits the total economic value of the technology adoption decision to reflect societal costs and benefits.

### A Bayesian value-based approach to designing a fixed sample size clinical trial

One of the key ideas underlying a value-based clinical trial is that the uncertainty surrounding a health technology assessment decision can be reduced by paying to recruit more patients to the trial. The presence of uncertainty means that there is a risk that better outcomes for patients, on average, could be achieved if an alternative technology adoption decision is made [20]. Reducing this uncertainty, by recruiting more patients, reduces this risk. This is because running the trial costs money, the ‘added value’ provided by recruiting additional patients and allocating them to the arms of the trial where they provide the maximum value should be compared with the cost of acquiring and retaining the patients, to judge their ‘value for money’ in reducing uncertainty.

Rooted in Bayesian decision theory, VoI analysis provides a framework for comparing the costs and benefits of running a fixed sample size clinical trial. Table 3 shows four levels at which VoI analysis can be conducted for such trials [34]. Evidence available before the start of the trial can be used to specify a ‘prior probability distribution’ for the unknown value of EINMB defined in Eq. (1). The prior probability distribution reflects the evidence available to the researchers at the start of the trial. Data accumulating during the trial are then used to update the prior distribution and obtain a ‘posterior distribution’ for the EINMB, using standard Bayesian methods [35]. The resulting expected value of the posterior distribution is a weighted average of prior information and the sampled data. Bayesian updating can take place on multiple occasions as the trial progresses, with multiple updates being made to the original prior distribution, giving a succession of posterior probability distributions.

**Table 3:** Four levels at which VoI analysis can be conducted for fixed sample size trials

Applied to the field of health technology assessment, value of information analysis is a Bayesian approach which compares the expected value of a decision made without collecting new information with the expected value after collecting, and acting upon, that new information. We can define four ideas:	
1.	Expected value of perfect information (EVPI): the value of acquiring perfect information about all aspects of the technology adoption decision (thereby eliminating all uncertainty) and acting accordingly;
2.	Expected value of partial perfect information (EVPPI): the value of acquiring perfect information about a subset of parameters in the decision and acting accordingly;
3.	Expected value of sample information (EVSI): the value of reducing, but not eliminating, the decision uncertainty by collecting information in a sample (e.g. by running a clinical trial) and acting accordingly;
4.	Expected net benefit of sampling (ENBS)—the EVSI minus the cost of acquiring the information (such as the cost of running the clinical trial)

Source: Raiffa and Schlaifer [34] and Fenwick et al. [20]. See also [37–39]

As more patients are recruited into the trial, the estimate of cost-effectiveness becomes more precise and this is reflected in the reduced variance in the posterior distribution for EINMB. Increased precision reduces the risk of making an incorrect decision about which health technology is superior on cost-effectiveness grounds, but it reduces the ‘value-added’ of recruiting an additional patient. Eventually, a point is reached at which the expected cost of recruiting another patient is equal to the expected benefit. This point defines the fixed sample size trial’s so-called ‘optimal’ sample size: prior to reaching the optimal sample size, the expected benefits of recruiting another patient outweigh the expected costs; beyond it, the expected costs outweigh the expected benefits.

This value-based approach has the advantage of incorporating parameters that are all meaningful from a clinical, medical and health policy standpoint because it includes measures of patients’ health outcomes, treatment costs, research costs, technology switching costs and the size of the population to benefit from the technology adoption decision, as well as the willingness to pay of the HTA agency for health gain. These measures reflect the increasing emphasis on delivering value to publicly funded health care systems. In particular, the design of the trial is not focused solely on estimating an incremental treatment effect at the level of an individual patient, nor is it governed by the traditional, frequentist, type I and type II error criteria. These might not adequately represent quantities such as disease prevalence, average health benefit and the incremental costs generated by the health technologies.

### The Bayesian value-based approach to designing an adaptive clinical trial

The Bayesian value-based approach to designing an adaptive clinical trial extends the value-based approach to designing a fixed sample size trial by allowing the trial’s accruing cost-effectiveness evidence to be compared with the cost of running the trial to inform changes to the trial as it progresses. The expected costs of continuing the trial in its current form versus changing it can be calculated and used to decide whether to change the trial or maintain the status quo.

If desired, simulations of value-adaptive trials can be run to produce estimates of frequentist power, bias and other characteristics, in line with published guidelines for reporting the characteristics of complex innovative trials [36].

In the next two sections, we illustrate an application of the value-adaptive approach to designing an adaptive clinical trial by reviewing the value-based design of a two-armed clinical trial with pairwise allocations to the arms proposed by Chick and collaborators [5, 7]. In Sect."The value-based sequential two-arm clinical trial design with adaptive sample size"we present an overview of the methodology and in Sect."Application of the Bayesian value-based sequential design to three published retrospective case studies"we summarise the results of three published applications of the model to retrospective data from UK clinical trials.

#### The value-based sequential two-arm clinical trial design with adaptive sample size

The value-based sequential clinical trial design proposed by Chick et al. 2017 [7] is a specific type of value-adaptive design. In this design, patients are randomised to one of two treatments and the trial’s stop/continue decisions are informed by collecting information on the accumulating estimate of individual and population EINMB, the number of patients whose outcomes have been observed (which determine the precision of the estimate of EINMB) and the expected costs of running the trial and of switching technologies.

The design assumes that follow-up of the cost-effectiveness data for each patient takes place after a defined period. This can be as small as a couple of hours, or as large as several years. For a value-based sequential design, the follow-up period must be smaller than the trial’s planned recruitment length so that, given the cost-effectiveness evidence accumulated at a given interim analysis, a ‘stop trial/continue trial’ decision makes sense; if the follow-up period is greater than the recruitment period, there is no opportunity to stop the trial before it reaches its maximum planned sample size.

The value-based sequential design uses the VoI methods described in Sect."Estimating the cost-effectiveness of a health technology", but within a dynamic framework. It uses what is termed a ‘dynamic programming’ approach to define the trial’s ‘stopping rule’ [7, 40–42]. The stopping rule halts recruitment of further patients when the expected benefit of continuing is not worth the expected cost. In this way, the overall expected value of the trial to the funder is maximised. The expected value is measured by the total net monetary benefit that patients expect to receive from the health technology assessment decision, less the cost of the research and, if relevant, of adopting one of the two technologies.

**Fig. 1:**
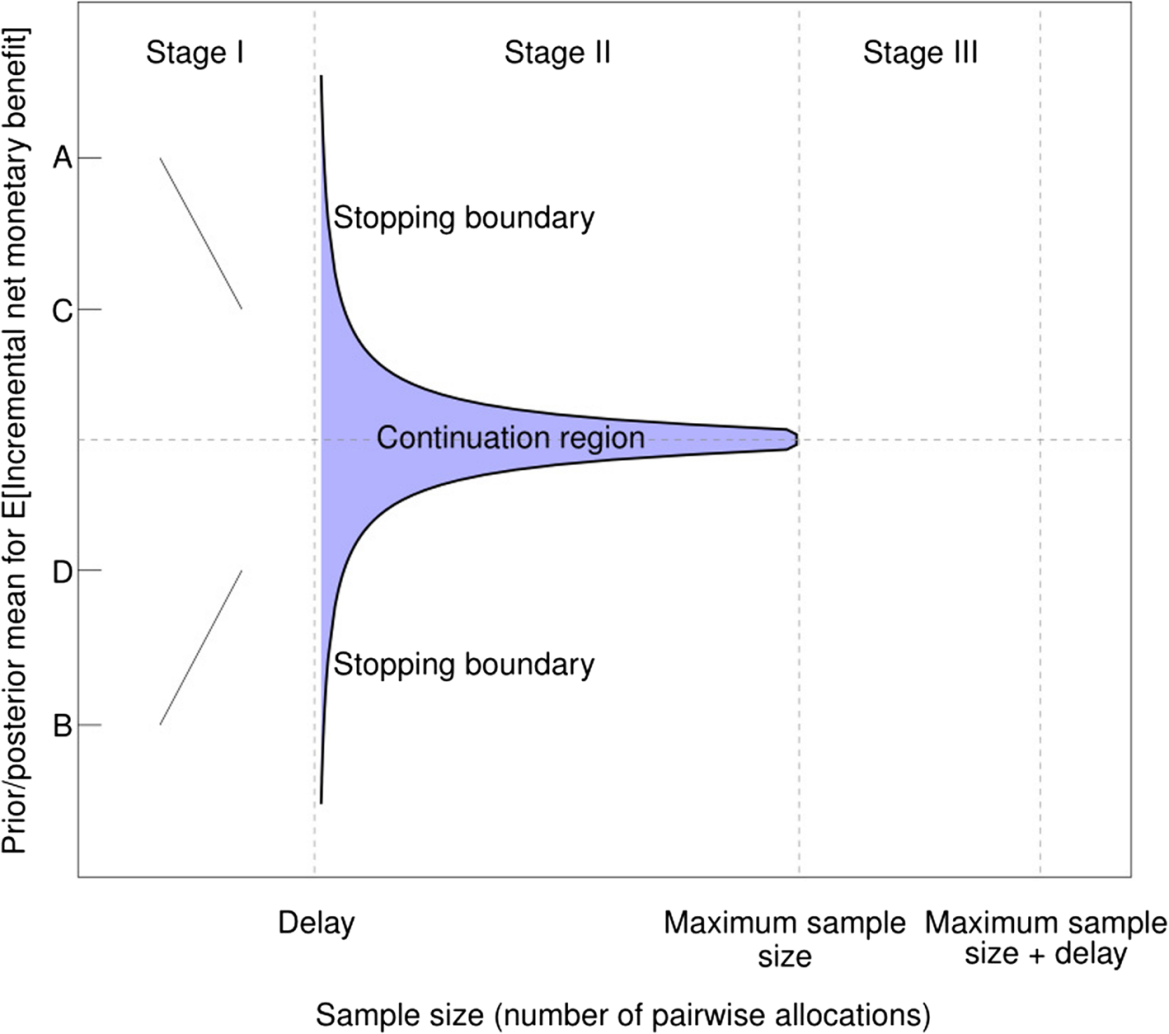
Illustration of a typical stopping boundary for the value-based sequential design described in Sect."The value-based sequential two-arm clinical trial design with adaptive sample size"(Note: in Stage I patients are recruited but no outcomes observed, In stage II patients continue to be recruited and outcomes from earlier patients are observed, as long as the statistics of the trial remain in the continuation region. The figure shows Stage III, when no patients are recruited but outcomes are still being observed, for the case when Stage II runs to its maximum sample size. If the trial stops prior to reaching its maximum sample size, Stage II finishes once the boundary is first crossed and Stage III starts earlier.)

The trial’s stopping rule is operationalised by defining a ‘stopping boundary’, which is best viewed graphically as in Fig. 1. The stopping boundary is obtained at the start of the trial and, as the trial progresses, the research team compares the expected value of the posterior distribution for EINMB after outcomes for *n* patients have been observed with the stopping boundary. If the posterior mean goes outside of the continuation region defined by the stopping boundary, recruitment to the trial halts and the remaining patients ‘in the pipeline’ – those who have been treated but whose outcomes are yet to be observed – are all followed up, prior to the adoption decision being made.

Figure 1 illustrates a typical stopping boundary for this kind of trial. The vertical axis displays the prior/posterior mean of EINMB. The horizontal axis shows the trial’s sample size, measured in terms of the number of pairs of patients recruited and randomised into the trial, up to the maximum sample size for the trial (marked ‘Maximum sample size’). The axis extends beyond this point to permit patient data to be monitored when there exists delay in observing the patient-level cost-effectiveness data. The figure shows that the design has three distinct stages:

1. During Stage I, patients are recruited to the trial and randomised to the two treatments, but cost-effectiveness data are not available until the defined follow-up point – labelled ‘Delay’ in Fig. 1 – is reached for the first pair of patients recruited to the trial. For example, if the time to follow-up of cost-effectiveness data is one year, Delay is equal to the number of pairs of patients recruited to the trial over one year.
2. During Stage II, outcome data and treatment cost data are accruing and are used to update the prior distribution for cost-effectiveness. There is the option to recruit and randomise more patients to the two arms of the trial, or to stop recruitment. The stopping boundary demarcates the region where the trial should continue to recruit patients (the shaded ‘continuation region’) from the region where the trial should stop. The shape of the stopping boundary and the size of the continuation region will depend on the trial-specific parameters that are used to solve the value-based sequential model. These include *P*, the variance of EINMB in the population (sometimes called the ‘sampling variance’), the time to follow-up of the cost-effectiveness data, the cost of sampling, the cost of switching technologies and the societal willingness to pay for one unit of health, such as a QALY.
3. During Stage III, recruitment to the trial has finished (either because the Stage II stopping boundary has been crossed or because the trial’s maximum planned sample size has been reached). Data from patients who were ‘in the pipeline’ when the trial stopped are observed and recorded as their time to follow-up is reached. When all data for all recruited patients have been observed, the technology that is estimated to be cost-effective, according to the criteria in Sect."Estimating the cost-effectiveness of a health technology", is considered for adoption.

As well as providing a rule for stopping the sequential trial using value-based criteria, the choice of prior mean for the trial may be used to choose the best kind of trial design from the following choices: run no trial at all; run a non-adaptive value-based design of the kind described in Sect."A Bayesian value-based approach to designing a fixed sample size clinical trial"; run the value-based sequential trial. These ideas are also illustrated in Fig. 1: if the prior mean is sufficiently high or low (that is, above A or below B in Fig. 1), the expected value of immediately adopting one of the two technologies exceeds the expected value of running any trial. This might happen, for example, if earlier-stage trial data were extremely favourable towards one of the two technologies, warranting an immediate adoption recommendation. For values of the prior mean between the points labelled C and D, it is optimal to run a value-based sequential design. For intermediate values– between points A and C or between B and D – it is optimal to run a design with a fixed sample size, selected by maximising the expected net benefit of sampling as described in Sect."A Bayesian value-based approach to designing a fixed sample size clinical trial".

#### Application of the Bayesian value-based sequential design to three published retrospective case studies

We review the application of the Bayesian value-based sequential design to three published case studies using retrospective clinical trial data from the United Kingdom: the ProFHER pragmatic trial, which was funded by the NIHR to compare surgical and nonsurgical intervention (sling immobilisation) for the treatment of proximal humerus fracture [44]; the CACTUS trial, which was funded by the NIHR and the North of Tyne PCT to evaluate the clinical and cost-effectiveness of a computer-based speech and language therapy (CSLT) in patients with aphasia following stroke [45]; the HERO trial, which evaluated whether hydroxychloroquine is superior to placebo for the treatment of hand osteoarthritis [46]. More detail about these analyses can be found in: for the ProFHER trial, Forster et al. [47]; for the HERO trial, Welch et al.[48]; for the CACTUS trial, Flight et al. [49]. Key features of the three trials are presented in Table 4. For all three trials, our analyses take the UK perspective. The three case studies were done at different times and built upon previous health economic evaluation work, with the willingness to pay for one QALY being different in the case studies but all within the typical threshold of £20,000 to £30,000 used by NICE [18] (£30,000 per QALY for ProFHER and HERO evaluations; the slightly lower £20,000 per QALY for CACTUS).

**Table 4:** Description of trials used for a case study analysis demonstrating how the value-based sequential design can be applied in practice

**The ProFHER pragmatic trial**
The ProFHER pragmatic trial was funded by the NIHR to compare surgical and nonsurgical intervention (sling immobilisation) for the treatment of proximal humeral fracture [44]. It was designed using a traditional, frequentist, approach which randomised 250 patients to the two arms of the trial, over the course of two and a half years. The trial cost £1.5 m and concluded that surgery was neither more effective than sling, nor more cost-effective, at two years’ follow-up. A follow-up at five years found that these results were unchanged [50]
**The Big CACTUS trial**
The Big CACTUS trial evaluated the clinical and cost-effectiveness of a computer-based speech and language therapy (CSLT) in patients with aphasia following stroke. The trial was funded by the NIHR Health Technology Assessment programme (HTA—12/21/01) with a budget of £1.4 m to cover research costs[51]. The trial used a traditional, frequentist approach that randomised 278 patients to three treatment arms. The long-term cost-effectiveness of the CSLT was assessed using a model based cost-utility analysis [52]. The trial showed that CSLT led to significant improvements in word-finding ability, but these did not generalise to conversation or patients’ perceptions of communication, participation and quality of life [52]. The cost-effectiveness analysis suggested that CSLT is unlikely to be considered cost-effective in the whole population investigated, but may be more cost-effective for people with mild to moderate word-finding abilities. Here, we focus the analysis assuming that two of the three arms were being analysed
**The HERO trial**
The HERO trial was a double-blind, randomised, clinical trial that evaluated whether hydroxychloroquine is superior to placebo for the treatment of hand osteoarthritis (OA). The study was funded by Arthritis Research UK (now Versus Arthritis UK) and had a budget of £900,000 [46]. Follow-up took place at six months for the clinical evaluation and at 12 months for the economic evaluation. The trial showed that hydroxychloroquine was no more effective than placebo for pain relief in patients with moderate to severe hand pain and radiographic osteoarthritis, nor was it found to be cost-effective [53]

For each application, we compare performance characteristics for three different clinical trial designs:

1. The original, frequentist, fixed sample size trial, designed according to traditional frequentist principles for power and Type I error probabilities and not value-based principles;
2. A Bayesian value-based one-stage design that maximises the expected net benefit of sampling, as described in Sect."A Bayesian value-based approach to designing a fixed sample size clinical trial";
3. The Bayesian value-based sequential design of Sect."The value-based sequential two-arm clinical trial design with adaptive sample size", with a maximum sample size chosen to be equal to the optimal sample size of the value-based one-stage design.

**Fig. 2:**
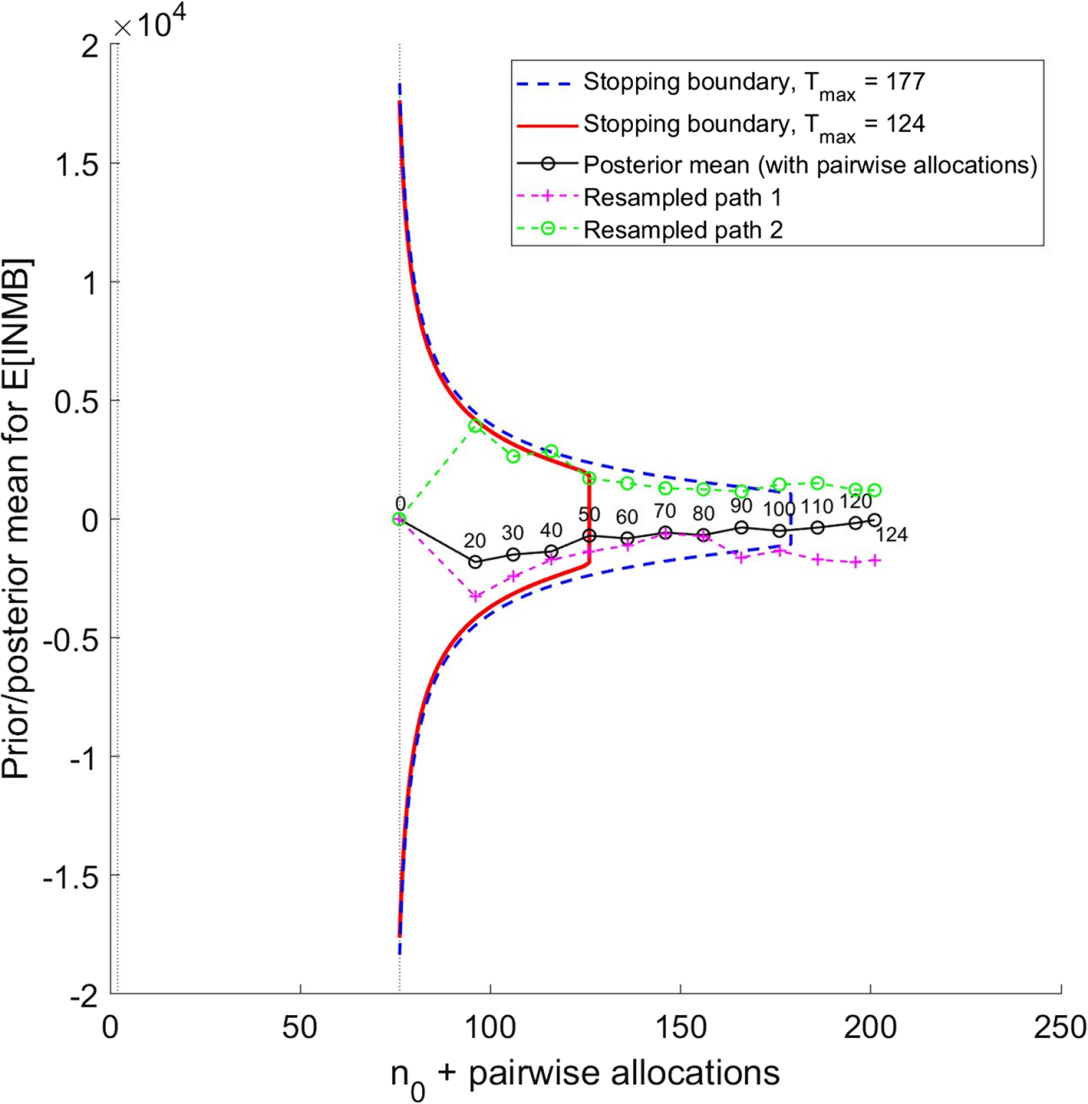
Stopping boundary for the value-based sequential two-arm design with a maximum sample size equal to: 1. the optimal sample size of the value-based one-stage design (177 pairwise allocations); 2. the sample size used in the HERO trial (124 pairwise allocations). Also shown is the path for the posterior mean from the HERO trial (black line, continuous) and two resampled paths (green and pink lines, discontinuous). Here, the effective number of samples in the prior distribution for the unknown INMB is n_0_ = 2

We assessed the performance of these designs by running Monte Carlo simulations based on 5000 bootstrapped samples for each simulated trial. To understand the general idea behind how these Monte Carlo simulations work, consider Fig. 2. This shows the stopping boundary for the value-based sequential design from the HERO application of Welch et al.[48], when the maximum sample size is equal to 124 pairwise allocations (red, continuous boundary; this is the sample size chosen for the HERO trial) and 177 pairwise allocations (blue, dashed boundary; this is the optimal sample size of the value-based one stage design). The trial data path for the posterior mean for the expected value of incremental net monetary benefit of hydroxychloroquine compared with placebo is shown in black, with interim analyses marked using small circles. Each circle is labelled with the number of pairwise allocations which contribute to the respective interim analysis, which we set at every ten pairwise allocations. No interim analysis took place after ten pairwise allocations owing to data sparsity (further details can be found in Welch et al. [48]).

Two bootstrap sample paths are also shown in Fig. 2. ‘Resampled path 1’ is a dashed, pink line and ‘Resampled path 2’ is a dashed, green line. The point at which each of the paths starts marks the start of Stage II of the sequential trial. The black and pink paths remain in the Stage II continuation region for a value-based sequential design whose maximum sample size is 124 pairwise allocations (red stopping boundary), meaning that if either of these two paths had been the path from the clinical trial, it would not have stopped early and would, instead, have run to the maximum sample size of 124 pairwise allocations. The green path crosses the upper stopping boundary between the interim analyses for 30 and 40 pairwise allocations, so if it had been the path from the trial, recruitment would have stopped at 40 pairwise allocations and the sample size of the trial would have been 114 pairwise allocations.

Once all pipeline data have been observed, the final points on both the black and pink paths are negative, meaning that hydroxychloroquine is estimated not to be cost-effective. Instead, the final point on the green path is positive, meaning that hydroxychloroquine is estimated to be cost-effective. For each of our three applications, the proportion of bootstrapped paths which show the new technology to be cost-effective is used as the estimate of the probability that the new technology is cost-effective. The trial’s expected sample size (measured in the number of pairs of patients recruited) is calculated by averaging the sample sizes of the bootstrapped paths; the expected cost of the trial is calculated by multiplying the average sample size by the estimated cost of randomising a pair of patients into the trial and adding the estimated fixed costs of the trial. Finally, the expected net benefit is calculated by multiplying the final value of the posterior mean for each path by the willingness to pay of the funder and the number of patients expected to benefit from the technology adoption decision and subtracting the estimated cost of the trial.

Table 5 summarises some of the operating characteristics from the bootstrap analysis of these trials. In all three case studies, it shows that the value-based sequential design delivers the highest expected net benefit for the healthcare system: the sample sizes for the original trial designs were not chosen according to value-based criteria, so it is no surprise that they deliver less value. The largest gain in expected net benefit of the value-based sequential design versus the original design is found for the CACTUS case study (+ 6.7%). Regarding the comparison of the non-adaptive value-based design with the value-based sequential design, the value-based sequential design offers the flexibility to stop the trial when the expected benefits of randomising a further pair of patients is not worth the cost, which is an option that is not available in the value-based one-stage design. The additional value generated by the value-based sequential design comes from this flexibility. For the ProFHER and HERO case studies this gain is very small, being less than 1%.

**Table 5:** Summary of operating characteristics for the three retrospective case-studies described in Sect."Implications of taking a value-adaptive approach in publicly funded research"

	**ProFHER case study (% difference from original trial)**	**CACTUS case study (% difference from original trial)**	**HERO case study (% difference from original trial)**
**Expected sample size (percent change in sample size relative to original trial)**			
Original trial	125	95	124
Value-based one-stage	112 (− 10%)	435 (+ 358%)	177 (+ 43%)
Value-based sequential	73 (− 42%)	166.01 (+ 74.7%)	174 (+ 40%)
**Expected cost associated with conducting the proposed trial design**			
Original trial	£1.47 m	£1.22 m	£0.84 m
Value-based one-stage	£1.42 m (− 3.4%)	£2.82 m (+ 130%)	£0.92 m (+ 10.5%)
Value-based sequential	£1.25 m (− 15%)	£2.66 m (+ 117%)	£0.92 m (+ 10.3%)
**Expected net monetary benefit**			
Original trial	£51.2 m	£102 m	£52.0 m
Value-based one-stage	£51.2 m (+ 0.01%)	£107.3 m (+ 5.3%)	£52.04 m (+ 0.01%)
Value-based sequential	£51.2 m (+ 0.27%)	£108.8 m* (+ 6.7%)	£52.14 m (+ 0.19%)

‘Original trial’ refers to the original (frequentist) fixed sample size design;

Value-based one-stage’ refers to the (non-adaptive) value-based design described in Sect."A Bayesian value-based approach to designing a fixed sample size clinical trial"

Value-based sequential’ refers to the value-based sequential two-arm design that is described in Sect."The Bayesian value-based approach to designing an adaptive clinical trial", with a maximum sample size equal to the sample size of the value-based one-stage design

(Sources: for ProFHER: Forster et al. [39]; for CACTUS: Flight et al. [49] and HERO: Welch et al. [48])

^*^calculated using htadelay package [56] rather than bootstrapping (see Flight et al. [49])

In the CACTUS case study, the optimal sample size of the value-based sequential design is 74.7% higher than the sample size of the original Big CACTUS trial. This greater sample size is due to the considerable residual uncertainty as to which arm is more cost-effective and the extra observations result in significant additional expected net benefit (+ 6.7% when compared with the original design of the trial). In contrast, for the HERO case study, the optimal sample sizes of the value-based one-stage design and value-based sequential design are 40–43% higher, but they deliver little additional expected net monetary benefit (less than 1%). The ProFHER case study value-based designs have a smaller expected sample size; however, they deliver only a small additional expected net monetary benefit.

The analysis for Table 4 was carried out under the assumption that the research costs of each trial, as actually incurred, could be used to inform the research costs of the value-based designs. However, it could be the case that the research costs of the value-based designs could differ. For example, extra data collection and analysis costs might accompany the more frequent interim analyses. Those costs should also be considered when choosing an appropriate design and would be straightforward to incorporate (we note that digital technology developments are reducing those costs through time).

### Making trials value-adaptive

The three case studies illustrate that, by taking appropriate care in choosing and valuing parameters which appropriately measure the overall value of health technologies and the clinical trial to the healthcare system, it is possible to obtain retrospective applications of a value-adaptive design using real-world data. It is likely that retrospective results such as these could be achieved for the other value-adaptive designs listed in Table 1 of this paper.

Unsurprisingly, the retrospective application results suggest that a value-adaptive design can deliver varying degrees of ‘value’ to the healthcare system, according to the strength of the cost-effectiveness signal that arises in the trial and the precise parameter values that apply to the health technology assessment. For example, had the value-based sequential trial been used instead of the traditional fixed sample design that was used in the ProFHER pragmatic trial, results suggest that the trial could have stopped earlier than planned with a sample size 42% lower than that used in the trial itself, reducing the time to adoption of the more cost-effective treatment and delivering a modest saving in research costs to the healthcare system. This result is primarily due to the strong cost-effectiveness signal favouring one of the two health technologies (non-surgical intervention) that emerged during the trial. In contrast, the weak cost-effectiveness signal from the HERO trial – the cost-effectiveness evidence suggested that hydroxychloroquine was shown to be little different to placebo for the treatment of hand osteoarthritis – suggests that a value-based sequential approach would not have led to earlier stopping.

There are many areas for future research in value-adaptive designs. The retrospective applications assume that there is a fixed size population to benefit from the treatment adoption decision. Alban et al. [5] illustrates how patent protection periods that decrease in the length of the trial affect the optimal fixed duration length of a value-based trial design. Expert elicitation techniques might be used to assess the requisite prior distributions [29, 54]. Pilot data and machine learning techniques could also be used to inform the choice of prior distribution, even for multi-arm value-adaptive trials [8]. Bias is a known issue in the analysis of adaptive trials [3]. It has been shown to have effects in health economic analysis of adaptive trials [55]. Existing corrections to adjust for the mean EINMB for the patients to be treated can be used in case the trial participant population differs somewhat from the population of patients to be treated post-adoption. Further work should consider how to incorporate bias adjustment of primary and secondary trial endpoints into the value-adaptive calculations.

One challenge for researchers in this field is to establish to what extent value-adaptive designs can deliver greater value to publicly funded healthcare systems through prospective application. It is likely that ‘increased application, experience and refinement’ of such methods is required to answer this question. One sensible ‘next step’ would be to use a value-adaptive design and use Monte Carlo simulation to generate power curves for the trial and to revisit how to make per patient trial costs or the size of the adopting population suitable to meet any relevant additional power curve constraints. Another option might be to use a value-adaptive design to ‘shadow’ a clinical trial which has been approved according to more traditional design criteria. This would allow a research team to run the value-based sequential design in the context of prospective data collection to understand the processes that need to be in place for a future clinical trial using solely the value-based design.

## Implications of taking a value-adaptive approach in publicly funded research

This section discusses some of the practical considerations for taking a value-adaptive approach in a publicly funded health system. These were developed as part of the ENACT project, in discussions with stakeholders from clinical trials units, research networks and the NIHR during two workshops in November 2019, through the experience of the ENACT collaborators who worked on the CACTUS and HERO case studies reported above and from feedback on the ENACT project report that was prepared for the NIHR [57].^3^ Reflecting the importance of accounting for cross-stakeholder views, as highlighted by the joint guidance from the NIHR and NHS England on “baking in” assessment of the value and real world cost of research as part of clinical research projects [58], we also considered the roles and activities of the following important stakeholder groups: research funders, trial research teams, patients and the public; Research Delivery Teams in health and care organisations, Health and Care Commissioners, HTA decision makers and clinicians.

Table 6 summarises some of the actions needed to implement value-adaptive designs. They are, classified into three stages: design and funding, conduct and analysis and reporting and implementation. Regarding the design and funding stage, by reflecting the overall objectives of the public healthcare system, a value-adaptive approach can provide useful guidance about the best design of the clinical trial, using the information that is available at the planning stage. For example, it can help guide a decision about whether it is worth making a technology adoption decision immediately, whether a value-based fixed sample size design is best or whether a value-adaptive design is preferable. The value delivered by the trial could be considered one of several important criteria for trial funding decisions, with others including (but not being limited to) fairness, access to, and exploration of, new health technologies [3, 15]. Stakeholders also noted that value-based designs could help inform analysis of the potential health gain and value delivered by different trial designs across intervention/disease areas, thereby prioritising research topics across a portfolio of studies in a particular disease area or funded by programmes such as the NIHR Health Technology Assessment funding stream [59–61, 63, 63].

**Table 6:**
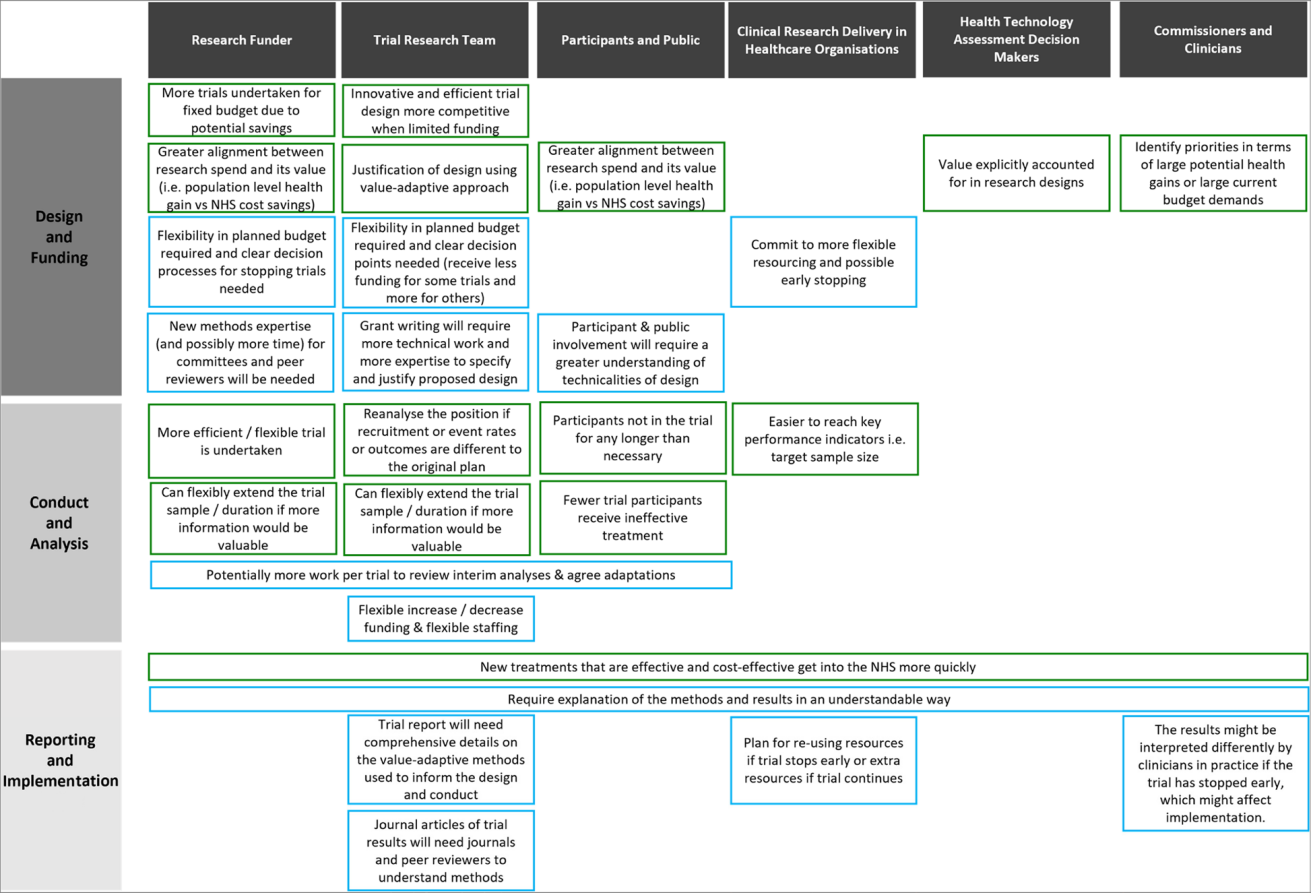
Mapping potential opportunities (green) & changes needed to action (blue) the value-adaptive approach in publicly funded clinical trials

The main opportunities arising at the conduct and analysis stage of a trial come in the flexibility of being able to amend the trial dynamically as data accrues, using the value-adaptive criteria described above. It was noted that more traditional, frequentist, adaptive designs already offer the flexibility to make amendments to the trial, using clinical effectiveness criteria alone, and that value-based methods could be assessed alongside traditional adaptive designs such as those using the O’Brien-Fleming or Pocock stopping rules [25, 63, 63]. Although discussions noted that a value-adaptive design is likely to cost more than a traditional trial design, owing to the added complexity from introducing interim analyses. These additional costs are likely to be mitigated if a value-adaptive design is used alongside a traditional, adaptive, clinical trial. The requirement for ongoing data monitoring to update the health economic outcomes would most likely build on existing data monitoring processes that already take place during the trial. There is a trade-off between these additional costs and the expected value of being value-adaptive: “How much value is your value-based method expected to create for me, over an existing design?” is an important question.

A further challenge, common to all Bayesian approaches (whether they are adaptive or not), is that it is necessary to obtain valid and justifiable prior distributions to describe the key existing uncertainties in the likely trial outcomes before the trial happens. This process has been well discussed in the literature [35]. The evidence to inform current uncertainty in outcomes could build on existing literature for the specification of prior distributions and come from expert elicitation processes [54], from relevant grey or archival literature, or data from a pilot study [63]. The research funding body could invest in funding for short pilot studies as part of providing such prior evidence.

Patients and public are an important stakeholder group and research teams might benefit from actively engaging patients and public in any value-adaptive design proposal early. As discussed by Flight et al. [25], value-based designs change the focus of clinical trials somewhat from the traditional clinical effectiveness viewpoint and the acceptability of this to the public and potential trial patients would need to be explored.

Regarding the reporting and implementation stage, as with all clinical trials research, methods and results should be reported in a transparent and understandable way for all stakeholders [63]. Clear sections reporting the value-adaptive approach would be necessary. To facilitate this, our ENACT project has added two case studies to the available worked examples, along with the open-source code [43, 48, 49].

Stakeholders also identified that planning communication to the clinical community by key opinion leaders could be important. This would include the methods, their use in practice and understanding relevant case study results and decisions. The work of the Health Innovation Network and analogous agencies in diffusing innovations in complex systems could be helpful [63].

Another challenge discussed around implementation concerned clinician familiarity with fixed sample size design trial evidence and it was discussed that this might generate some reluctance to implement an intervention based on findings from a value-adaptive design that terminates earlier than a fixed design might have done. Appropriate training and understanding of the methods should mitigate this.

A strength and limitation of our ENACT study programme was its strong focus on publicly funded research and hence little detailed discussion of commercially funded research. Much of the methodology and statistical approach could apply to commercially funded research and work would be needed to consider how commercial companies’ objectives could be incorporated, building on initial work that allows collaborative bargaining of prices as a function of health value created [32].

### Potential activities towards implementing value-adaptive designs in publicly funded research

Stakeholders were also asked to consider the activities and actions which could be useful or needed to enable implementation of value-adaptive designs (see Table 7). The suggestions reflect many of the recommendations reported by Blagden et al., in relation to the effective delivery of complex and innovative clinical trial designs [63] and also the work of Jaki and Dimairo et al., who considered why uptake of adaptive approaches has lagged behind their methodological development [63, 63].

**Table 7:**
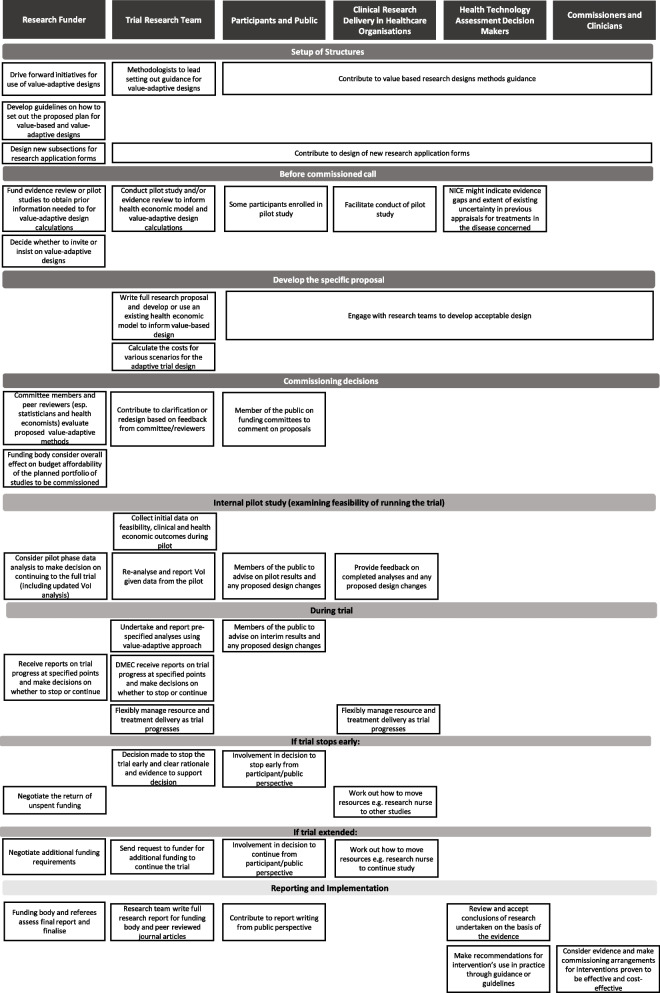
Mapping of the potential activities of NIHR stakeholders to move to implementing these methods during the healthcare research and decision-making process

Research funders are in a strong position to support initiatives for greater use of value-adaptive designs. One way to encourage this would be to include an indication in calls that funders would welcome trial applications which included a value-adaptive perspective. For example, a funding call could focus on projects which develop a pilot study to inform prior probability distributions for use in the value-adaptive design.

Research funding bodies might also provide guidance for researchers on how to set out the plans for a value-adaptive trial in research proposals. Activities in reporting progress to the funding body would build on existing processes and infrastructure. The trial team would need to undertake and report pre-specified analyses. An independent group, usually the data monitoring and ethics committee (DMEC) would still need to receive reports on trial progress and help to make recommendations on study adaptations to the trial team and funder. When the trial concludes, the DMEC, trial management group and trial steering committee would need to agree and approve the final analysis and the reasoning for criteria to end the trial given the available evidence. Stakeholders believed that the final reporting of trial results and the use of evidence in health technology assessment and clinical commissioning would have very little difference from current practice. The full research report for the funding body and the associated peer reviewed journal articles would follow usual procedures.

Finally, it was considered important that patients, clinicians, funders and health technology assessors can understand the approaches taken, to critically assess them and to interpret the results. This understanding could benefit from careful presentation by research teams including standardised reporting of relevant aspects, which could well be facilitated, for example, by the Adaptive Designs CONSORT Extension [63].

## Conclusions

Value-adaptive approaches to clinical trial design provide a range of novel techniques to improve the societal value of clinical trials by seeking to improve the expected learning for trial budgets relative to population health goals. This can include stopping the trial early, running the trial longer, changing the fraction of patients allocated to the arms, or making other adaptations which align better the current value-for-money trend in healthcare delivery with that of the design of the trial. This paper sets out the key methods involved, summarises the methods and results from three case studies and assesses the opportunities and challenges which arise for publicly funded research using the UK NIHR as an exemplar. Many of the systems to deploy value-adaptive designs already exist and some refinement to processes are likely to be needed. Increased experience and application of these methods will be useful on the pathway to implementation of the value-adaptive design approaches which offer potential for more efficient publicly funded health research.

## Data Availability

No datasets were generated or analysed during the current study.

## Appendix

The following information is required to implement the value-based sequential design:

1. the reimbursement agency’s willingness to pay threshold (discussed above);
2. the fixed and variable costs of carrying out the research;
3. the size of the population which is expected to benefit from the health technology assessment decision;
4. the sampling variance of the incremental net monetary benefit in the population of patients considered;
5. the expected value and variance of the prior distribution for the expected incremental net monetary benefit;
6. the follow-up period, defined as the period of time between randomising a patient to one of the two arms of the trial and observing the measure of incremental net monetary benefit for each patient;
7. any ‘switching cost’ that is expected to be incurred if a decision is made to switch from the existing technology to the new one.

Techniques to inform the prior distribution for the unknown expected incremental net monetary benefit of the new versus existing technology include subjective probability elicitation [54] and empirical Bayes methods using pilot data [63]. Performance characteristics explored by Forster et al. [47] include the expected sample size of the trial, its expected cost, the overall expected value delivered to the healthcare system by the trial and the probability that the trial concluded that one of the two treatments was cost-effective.

To estimate these characteristics before the trial begins, a trial team can use the software provided by Chick et al. [56] (available from https://github.com/sechick/htadelay) and pre-trial simulations. The actual data accrued during a trial represent just one realisation of how the trial data emerge. Pre-trial simulations can be used to model how the data might arrive in a different order many times. As the case studies listed in Table 6 were retrospective, we used a non-parametric bootstrapping analysis to simulate possible trial results, as the original trial data were available. In practice, alternative approaches might be used such as parametric bootstrap approaches [63].

## Abbreviations

CONSORT: Consolidated Standards of Reporting Trials
CRN: Clinical Research Network
DMEC: Data Monitoring and Ethics Committee
ENACT: EcoNomics of Adaptive Clinical Trials
EINMB: Expected Incremental Net Monetary Benefit
ENBS: Expected Net Benefit of Sampling
EVPI: Expected Value of Perfect Information
EVPPI: Expected Value of Partially Perfect Information
EVSI: Expected Value of Sample Information
HTA: Health Technology Assessment
INMB: Incremental Net Monetary Benefit
NIHR: National Institute for Health and Care Research
NHS: National Health Service
NICE: National Institute for Health and Care Excellence
NMB: Net Monetary Benefit
QALY: Quality Adjusted Life Year
UK: United Kingdom
UKRI: UK Research and Innovation
VoI: Value of Information
WTP: Willingness to Pay

## Footnotes

1 Methodological innovations from the NIHR are often adopted by the Association of Medical Research Charities (AMRC) and UK Research and Innovation (UKRI), which plays an important part in the UK non-commercial research sector. Similar public funders exist in other countries with developed healthcare systems, including Canada (Canadian Agency for Drugs and Technologies in Health. Guidelines for the Economic Evaluation of Health Technologies: Canada. 2017. https://www.cadth.ca/guidelines-economic-evaluation-health-technologies-canada-0) and Australia (Pharmaceutical Benefits Advisory Committee. Guidelines for preparing submissions to the Pharmaceutical Benefits Advisory Committee. Version 4.3. 2008. https://pbac.pbs.gov.au/), making the findings relevant internationally.

2 Although the QALY is one of the most common health outcomes that is used to provide a monetary valuation, it is not the only one. For example, Meltzer et al. (2011) used a monetary estimate of a day free from sinusitis in their study of the impact of antibiotics on the average time to recovery from acute bacterial sinusitus.

3 We structured these workshop discussions around the three stages of clinical trials research identified in the “NIHR Clinical Trials Toolkit Route map” [63]. Stage 1, Design and Funding, covers trial planning and design, funding proposal development, funding panel review; Stage 2, Conduct and Analysis, includes protocol and trial documentation development, ethics approval, internal pilot, trial management and monitoring, safety monitoring, statistical analysis, health economic analysis, and monitoring by funders; Stage 3, Reporting and Implementation, covers reporting results, health technology assessment and implementation of proven interventions into clinical practice.
